# Modulation of the Fibrillation Kinetics and Morphology of a Therapeutic Peptide by Cucurbit[7]uril

**DOI:** 10.1021/acs.molpharmaceut.3c00185

**Published:** 2023-06-16

**Authors:** Marcello Martinez Morales, Christopher F. van der Walle, Jeremy P. Derrick

**Affiliations:** † Dosage Form Design & Development, AstraZeneca, Aaron Klug Building, Granta Park, Cambridge CB21 6GH, U.K.; ‡ School of Biological Sciences, Manchester Academic Health Science Centre, The University of Manchester, Manchester M13 9PL, U.K.

**Keywords:** enfuvirtide, cucurbit[7]uril, fibrillation, kinetics, amyloid

## Abstract

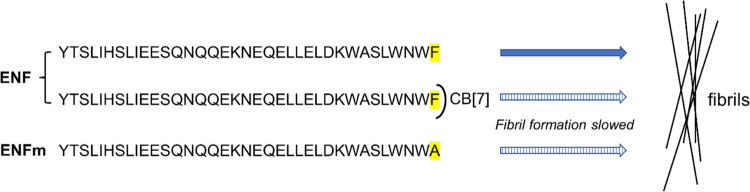

Fibrillation is a challenge commonly encountered in the formulation and development of therapeutic peptides. Cucurbit[7]urils (CB[7]), a group of water soluble macrocycles, have been reported to suppress fibrillation in insulin and human calcitonin through association with Phe and Tyr residues which drive fibril formation. Here, we report the effect of CB[7] on the fibrillation behavior of the HIV fusion inhibitor enfuvirtide (ENF) that contains N-terminal Tyr and C-terminal Phe residues. Thioflavin T fluorescence, CD spectroscopy, and transmission electron microscopy were used to monitor fibrillation behavior. Fibrillation onset showed a strong pH dependency, with pH 6.5 identified as the condition most suitable to monitor the effects of CB[7]. Binding of CB[7] to wild-type ENF was measured by isothermal titration calorimetry and was consistent with a single site (*K*_a_ = 2.4 × 10^5^ M^–1^). A weaker interaction (*K*_a_ = 2.8 × 10^3^ M^–1^) was observed for an ENF mutant with the C-terminal Phe substituted for Ala (ENFm), suggesting that Phe was the specific site for CB[7] recognition. The onset of ENF fibrillation onset was delayed, rather than fully suppressed, in the presence of CB[7]. The ENFm mutant showed a greater delay in fibrillation onset but with no observable effect on fibrillation kinetics in the presence of CB[7]. Interestingly, ENF/CB[7] and ENFm fibrils exhibited comparable morphologies, differing from those observed for ENF alone. The results indicate that CB[7] is capable of modulating fibrillation onset and the resulting ENF fibrils by specifically binding to the C-terminal Phe residue. The work reinforces the potential of CB[7] as an inhibitor of fibrillation and highlights its role in determining fibril morphologies.

## Introduction

Peptides are capable of self-associating into ordered structures termed fibrils through a process known as fibrillation.^1^ While fibrils form naturally in secretory glandules functioning as a storage for peptide hormones, fibrillation is also linked to many neurodegenerative pathologies such as Alzheimer’s and Parkinson’s disease.^2,3^ Similarly, fibrillation is an unwanted phenomenon arising during the development of peptide drugs in the pharmaceutical industry.^4^ A body of literature exists about the causes of protein self-association, detailing underlying mechanisms at a molecular level and presenting strategies to mitigate aggregation propensity, while research output dedicated to formulation development of therapeutic peptides has only recently picked up.

One of the primary aims of peptide formulation development is to maintain peptide physical stability; fibrillation represents one of the main processes that requires control.^5^ Methods commonly used to characterize the fibrillation process include Thioflavin T assays (ThT-assays) and far-UV circular dichroism spectroscopy (FUV-CD) to monitor fibrillation progress and associated changes in the secondary structure, while imaging techniques such as transmission electron microscopy (TEM) and atomic force microscopy (AFM) are used to verify the presence of fibrils and examine morphological and structural features.^6^ In general, the fibrillation process is characterized by a lag phase (formation of critical nucleus), a growth phase (exponential growth), and saturation (monomer depletion or competing growth and dissociation processes), resulting in distinct fibril morphologies.^7^ The lag phase represents one of the critical quality attributes during peptide formulation and earlier studies have focused on identifying solution conditions that suppress or prolong fibrillation onset.^8^ Fibrillation onset is a function of several extrinsic factors such as solution pH, temperature, peptide concentration, and excipients, as well as intrinsic factors such as peptide sequence and propensity to form secondary structure motifs.^9^ For example, the fibrillation kinetics of glucagon-like peptide-1 (GLP-1) have been reported to follow different fibrillation pathways in a pH-dependent manner.^10,11^ Likewise, fibril morphologies have been observed to differ as a function of both intrinsic and extrinsic factors.^12−15^ Inhibition of fibrillation in therapeutic peptides has previously been achieved through the addition of excipients, such as surfactants.^16,17^ At a molecular level, aromatic amino acid residues capable of forming π-stacking interactions are believed to play an important role in fibrillation.^18^ Macrocyclic compounds able to bind aromatic residues such as cyclodextrins, calixarenes, and cucurbiturils have been reported to modify fibrillation behavior by binding to fibrillation-driving residues.^19−22^

**Figure 1:**
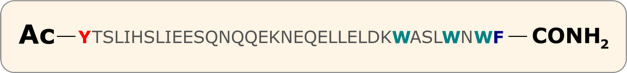
ENF sequence with aromatic side chains highlighted where CB[7] can potentially bind. The commercially available peptide, used in the present study, is acetylated at the N-terminus and amidated at the C-terminus. The terminal Phe residue is mutated to Ala in the ENFm mutant.

Enfuvirtide (ENF; also called Fuzeon, T-20 or DP-178) is the first commercially available HIV fusion inhibitor and was approved back in 2003^23^ (Figure 1). Its mechanism of action has been studied in detail.^24,25^ Commercially available ENF is formulated in a sodium carbonate buffer at a pH of around 9, with the final drug product being a lyophilized powder that is reconstituted prior to administration via subcutaneous injection.^26,27^ The high pH formulation is required because ENF is poorly soluble under acidic conditions.^28^ In addition, ENF suffers from a short plasma half-life of roughly 2 h, requiring two daily injections which can result in a reduction in patient compliance.^29^ Previous studies have mainly focused on examining the solubility and solution structure of ENF in different solution conditions.^30−32^ Although fibrillation can take place during manufacturing and storage, fibril formation of ENF has not been studied in detail to the best of our knowledge.

Cucurbit[7]uril (CB[7]) is a 7-membered macrocycle and a member of the cucurbit[*n*]uril (CB[*n*]) family consisting of glycouril units connected via methylene bridges. They form a hydrophobic pocket which, in the case of CB[7], is able to harbor aromatic amino acids, particularly Phe.^33^ This property can be usefully exploited in reducing aggregation, by binding to specific residues within surface aggregation-prone regions on the surface of a monoclonal antibody, for example.^33^ Two previous studies of the effect of CB[7] on fibrillation inhibition have been reported, although not on ENF.^21,34^ Moreover, the effect of CB[7] more generally on fibril morphology has not been investigated. Here, we look at the effect of CB[7] on the fibrillation behavior of ENF by examining fibrillation kinetics and fibril morphology in the absence and presence of CB[7]. The study also establishes the importance of a C-terminal Phe residue in ENF by examining the fibrillation kinetics and fibril morphology of a single-point mutant (ENFm) lacking the putative CB[7] binding site.

## Experimental Section

### Materials and Sample Preparation

Enfuvirtide (ENF) and the enfuvirtide mutant (ENFm) were purchased from Chinese Peptide Company (Hangzhou, China) as lyophilized powders (96.9 and 95.1% purity, respectively, as measured by RP-HPLC). Sodium phosphate monobasic and sodium phosphate dibasic were purchased from J. T. Baker (Avantor Performance Materials B.V., Arnhem, The Netherlands). Cucurbit[7]uril was purchased from Strem Chemicals UK (Cambridge, U.K.). Thioflavin T was purchased from Sigma-Aldrich (Poole, U.K.). 10 mM sodium phosphate buffer solutions at pH 6, 6.5, 7, and 7.4 were prepared with deionized water (18.2 MΩ, Milli-Q system, Merck-Millipore, Watford, U.K.). Peptide concentrations were determined using a NanoDrop (Thermo Fisher Scientific, U.K.) UV/vis spectrophotometer and theoretically calculated extinction coefficients of 18 350 M^–1^ cm^–1^ at 280 nm. A 5 mM CB[7] stock solution was prepared in a 10 mM sodium phosphate buffer at pH 6.5. ENF and ENFm were both dissolved in 10 mM sodium phosphate buffer, and a decrease in solution pH observed upon dissolution was corrected by addition of a few μL of a 50 mM NaOH solution to achieve pH values of 7.4, 7, 6.5, and 6 (volume of NaOH added varied depending on target pH). Subsequently, samples were exhaustively dialyzed (three buffer exchanges after 2, 4 and 6 h, each under gentle stirring for ∼24 h following the manufacturer’s instructions) using 0.1–0.5 kDa Spectra/Por Micro Float-A-Lyzer dialysis devices (Spectrum Laboratories Inc., Sancho Dominguez, CA). All solutions were filtered through a 0.22 μm filter prior use.

### Thioflavin T Fibrillation Assay

Thioflavin T assays were performed on a Fluostar Optima Microplate Reader (BMG Labtech, Offenbach, Germany) at a temperature of 37 °C. ENF samples of 40, 60, and 80 μM and ENFm samples of 80 μM were prepared as stated above. Thioflavin T was added to 50 μM concentration in the final samples. For the fibrillation assay, 120 μL of ENF or ENFm containing Thioflavin T was loaded into a Corning #3881 96-well half area plate made of black polystyrene with a clear bottom and a nonbinding surface (Corning Inc., Kennebunk, ME). The outer wells and space between wells were filled with water, and each plate was covered using sealing tape to avoid sample evaporation. Initial ThT-assays for ENF at different concentrations were run for a total of 280 cycles measuring fluorescence every 30 min. ThT fluorescence in the presence of CB[7] was measured for a total of 290 cycles every 10 min for ENF and for 220 cycles every 35 min for ENFm. Each measurement recorded 5 flashes per cycle and well, with a gain of 1000 and a 5 min orbital shaking interval prior to each measurement. Excitation and emission filter were set to 440 and 480 nm, respectively. Data was fitted using MATLAB and Statistics Toolbox Release R2018b (The MathWorks, Inc., Natick, MA) to the empirical equation
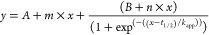
1with *A* representing the starting fluorescence, *m* representing the initial slope, *B* representing the fluorescence at the final baseline, *n* representing the slope at the final baseline, *t*_1/2_ representing the half-time, defined as the time at which the Thioflavin T fluorescence reaches 50% of the final baseline fluorescence and *k*_app_ representing the apparent growth rate.^11^ The lag time (*t*_lag_) was calculated as follows
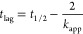
2All measurements were made in triplicate and repeated independently for three times.

### Far-UV CD Spectroscopy (FUV-CD)

Far-UV circular dichroism spectra of ENF and ENFm in the presence and absence of CB[7] were recorded on a Jasco J-815 spectrophotometer. Samples were taken at 4 different time points for ENF (0, 15, 30, and 45 h) and ENFm (0, 40, 80, and 120 h) during ongoing Thioflavin T assays. Peptide and buffer concentrations were as detailed in the previous section. Samples were transferred onto a 0.01 cm pathlength quartz cuvette (Starna Scientific Ltd., Hainault, U.K.) and measured from 190–250 nm at a scan rate of 60 nm/min at 25 °C. Recorded spectra from buffer were subtracted from each measurement and subsequently deconvoluted using the BeStSel webtool.^35,36^

### Negative-Stain Transmission Electron Microscopy (TEM)

Samples of ENF and ENFm in the presence and absence of CB[7] were adsorbed onto freshly glow—discharged 400 mesh copper/carbon-coated grids (EM Resolutions Ltd, Saffron Walden, U.K.) for 1 min. TEM grids were washed twice in 10 μL droplets of deionized water to remove any buffer salts. Subsequently, TEM grids were stained with a 10 μL droplet of 2% (w/v) aqueous uranyl acetate for 30 s. Uranyl acetate dye was drained from the TEM grids using filter paper and grids were allowed to air dry. Samples were viewed using a Tecnai G20 transmission electron microscope (FEI/Thermo Fisher Scientific, Loughborough, U.K.) run at an accelerating voltage of 200 keV using a 20 μm objective aperture to improve contrast.

### Isothermal Titration Calorimetry

Thermograms were recorded on a Microcal Auto-ITC_200_ (Malvern Panalytical Ltd., Malvern, U.K.). CB[7] was dissolved in the bulk buffer solution from the final dialysis of ENF and ENFm separately to avoid buffer mismatch. The measurement cell was loaded with 100 μM peptide, and 4 mM CB[7] was loaded into the syringe as the titrant. A prerinse with bulk buffer solution of the final dialysis was performed automatically before each titration. The titration consisted of 19 injections of 2 μL each, spaced by 120 s intervals and preceded by a single injection of 0.4 μL. The jacket temperature was set to 25 °C, and the stirring speed was set to 750 rpm. Thermogram processing and integration to obtain binding isotherms were performed in Origin Data Analysis 7.21 (Malvern Panalytical Ltd., Malvern, U.K.). Integrated heat resulting from the first injection of 0.4 μL was excluded from analysis. Integrated heat values obtained through titration of CB[7] into buffer solution were subtracted from experiments using point-by-point subtraction.

## Results

### Thioflavin T (ThT) Fluorescence of ENF as a Function of pH and Concentration

ThT fluorescence of ENF was examined at a range of concentrations and pH values to identify suitable solution conditions for monitoring ENF fibrillation behavior. Lyophilized ENF was solubilized in a 10 mM sodium phosphate buffer at pH 7.4 but required dialysis to remove trifluoroethanol (TFA) and avoid a reduction in pH. ThT fluorescence was monitored over time at 37 °C. The different phases of fibrillation (lag phase, exponential growth phase, and saturation) can be followed measuring ThT fluorescence, which typically results in a sigmoid-shaped profile.

**Figure 2:**
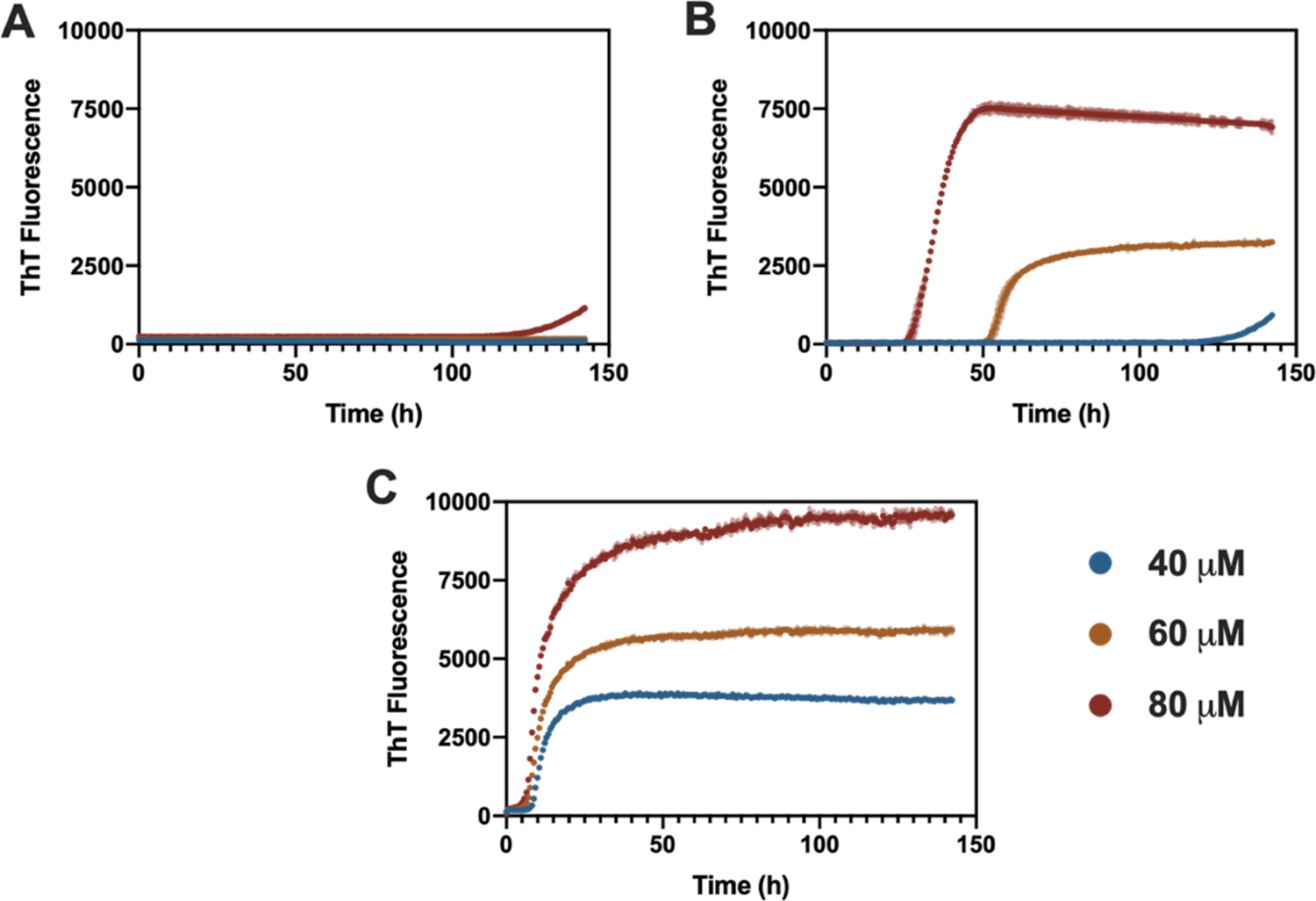
ThT-assays performed for ENF at pH 7 (A), 6.5 (B), and 6 (C). Assays were run for a total of 5 days at a temperature of 37 °C with samples at concentrations of 40, 60, and 80 μM and a ThT concentration of 50 μM. Results show the average of 3 replicates.

An initial assessment at pH 7.4 yielded no significant changes in ThT fluorescence up to an ENF concentration of 100 μM (Figure S1). pH conditions were therefore reduced to 7, 6.5, and 6, moving toward the theoretical pI of ∼4 for the peptide. ThT fluorescence under these conditions is shown in Figure 2, at ENF concentrations of 40, 60, and 80 μM. At pH 7, an increase in ThT fluorescence can only be observed at a concentration of 80 μM. However, the experiment duration is too short to obtain a typical fibrillation profile. This profile can be observed at pH 6.5 at concentrations of 60 and 80 μM and at pH 6 at all concentrations. The maximum measured fluorescence (Fl_max_) increases with ENF concentration at all pH conditions where a significant change in fluorescence can be observed. Furthermore, following ThT fluorescence at different pH values shows a clear pH dependency of fibrillation onset which shortens as the pH moves from neutral to slightly acidic conditions. The ThT profile of ENF at pH 6.5 and a concentration of 80 μM differs from the one at 60 μM and all profiles at pH 6 in that the saturation phase is marked by a downward slope. This suggests a mechanistic difference during the fibrillation process and presence of a secondary fibrillation pathway.

An empirical function (eq 1) representing an estimate of the kinetic reaction scheme involving a lag phase, nucleation, and saturation was used to extract kinetic parameters *t*_lag_ and *k*_app_. This was done for ThT fluorescence profiles at pH 6.5 and at ENF concentrations of 60 and 80 μM (Table 1). Here, *t*_lag_, describing the duration of the lag phase before significant changes in fluorescence can be observed, decreases as ENF concentrations increase from 60 to 80 μM, with fibril growth becoming slightly slower at higher ENF concentrations.

**Table 1:** Lag Time (*t*_lag_) and Apparent Growth Rate (*k*_app_) of ENF Fibrillation at pH 6.5

concentration (μM)	*t*_lag_ [h]	*k*_app_ [h^–1^]
60	51.4 (±1.4)	0.36 (±0.01)
80	27.5 (±0.5)	0.28 (±0.02)

The relationship between the derived *t*_lag_ and concentration is kinetically best described by a classical nucleation–polymerization model (NP), which requires formation of a nucleus of critical size before exponential fibril growth occurs.^7^ Additionally, changes of *t*_lag_ at different solution conditions indicate a strong dependency of fibrillation on solution pH. While samples at all concentrations at pH 7 did not achieve fluorescence saturation after an incubation time of 6 days, samples of all concentrations at pH 6 yielded typical sigmoidal profiles with fibril growth having started within the first 7 h of the assay and reaching saturation in less than 3 days.

A solution pH of 6.5 and a concentration of 80 μM were selected because the conditions yield a full fibrillation profile in less than 3 days, which is characterized by a well-defined baseline and plateau required for successful fitting and calculation of kinetic parameters.

### Structural Changes during ENF Fibrillation

β-sheets form the dominant secondary structure motif in mature fibrils.^1^ In previous reports, the ENF structure has been studied using FUV-CD; it was shown to be mainly unstructured in aqueous solution at physiological pH.^30,31^ Here, we monitored relative changes in the solution structure by FUV-CD in parallel with the ThT-assays. FUV-CD has previously been used to semiquantitatively monitor transitions toward β-sheet motifs.^1^

**Figure 3:**
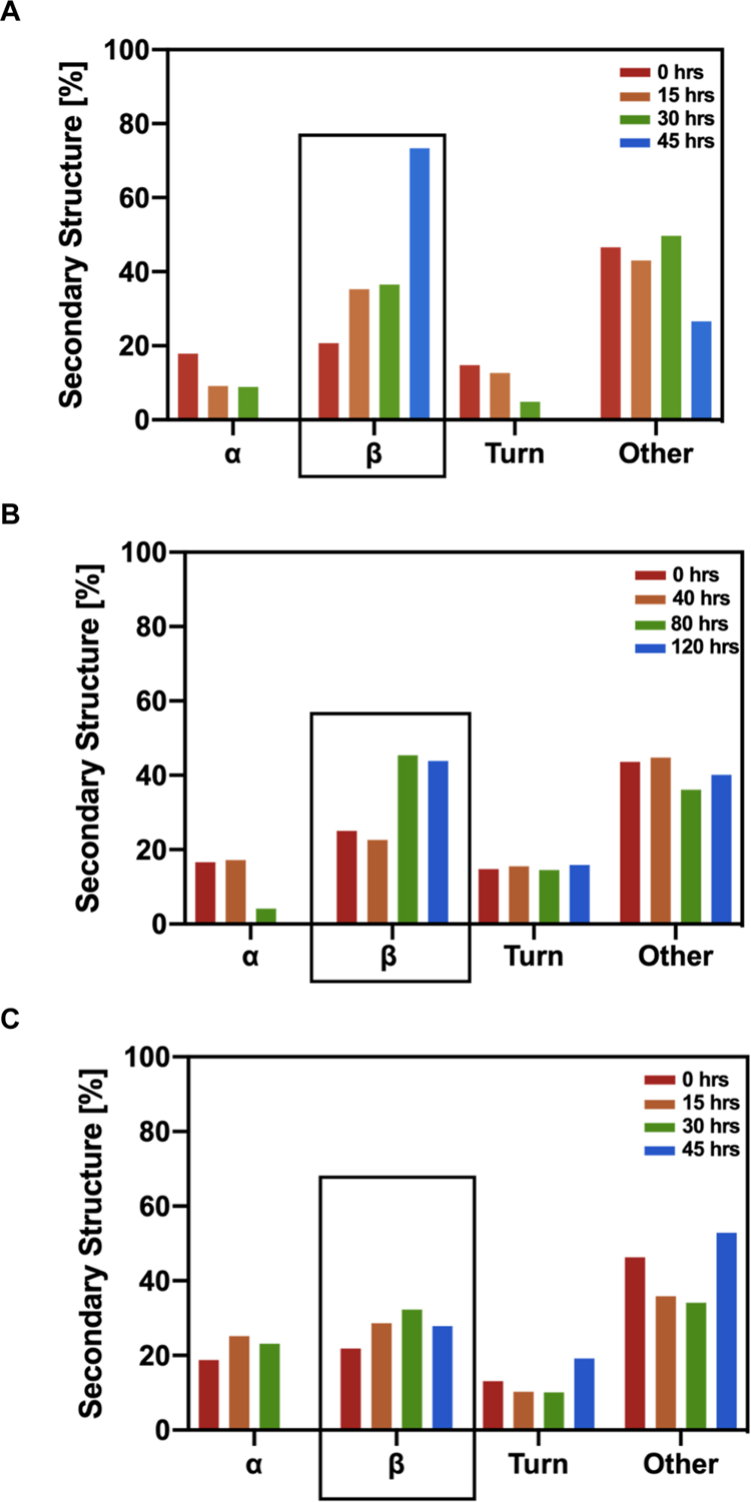
Secondary structure deconvolution of ENF and ENFm. (A) Secondary structure of ENF before and during a 45 h incubation at 37 °C. (B) Secondary structure deconvolution of ENF in the presence of CB[7] before and during a 45 h incubation at 37 °C. (C) Deconvolution of CD spectra of ENFm at different time points during fibrillation. Secondary structure was calculated using the BeStSel server, and the rectangle highlights changes in β-sheet contributions characteristic of mature fibrils.

Figure S2A shows CD spectra recorded for ENF before and during fibrillation. While CD spectra of α-helices have characteristic minima at 202 and 222 nm, those of β-sheets exhibit a single minimum in the region of 215–220 nm.^37^ Before incubation, ENF has a minimum at 202 nm and a slight shoulder in the region of 220 nm, suggesting that no well-defined secondary structure motif dominates. As time progresses during the incubation, the spectrum transitions to a well-defined minimum at 218 nm, indicating significant contribution of the β-sheet structure. Secondary structure contributions were calculated using the BeStSel server and are shown in Figure 3A.^35,36^ At *T*_0_, ENF remains predominantly unstructured (49.7%, red bar in Figure 3) with α-helical and β-sheet contributions to the overall structure. During the course of the incubation, random coil contributions to structure persist but with the β-sheet structure increasing up to 73.4% after 45 h, characteristic of mature fibrils.^38^

### Electron Microscopy Analysis of ENF Pre- and Post-Fibrillation

**Figure 4:**
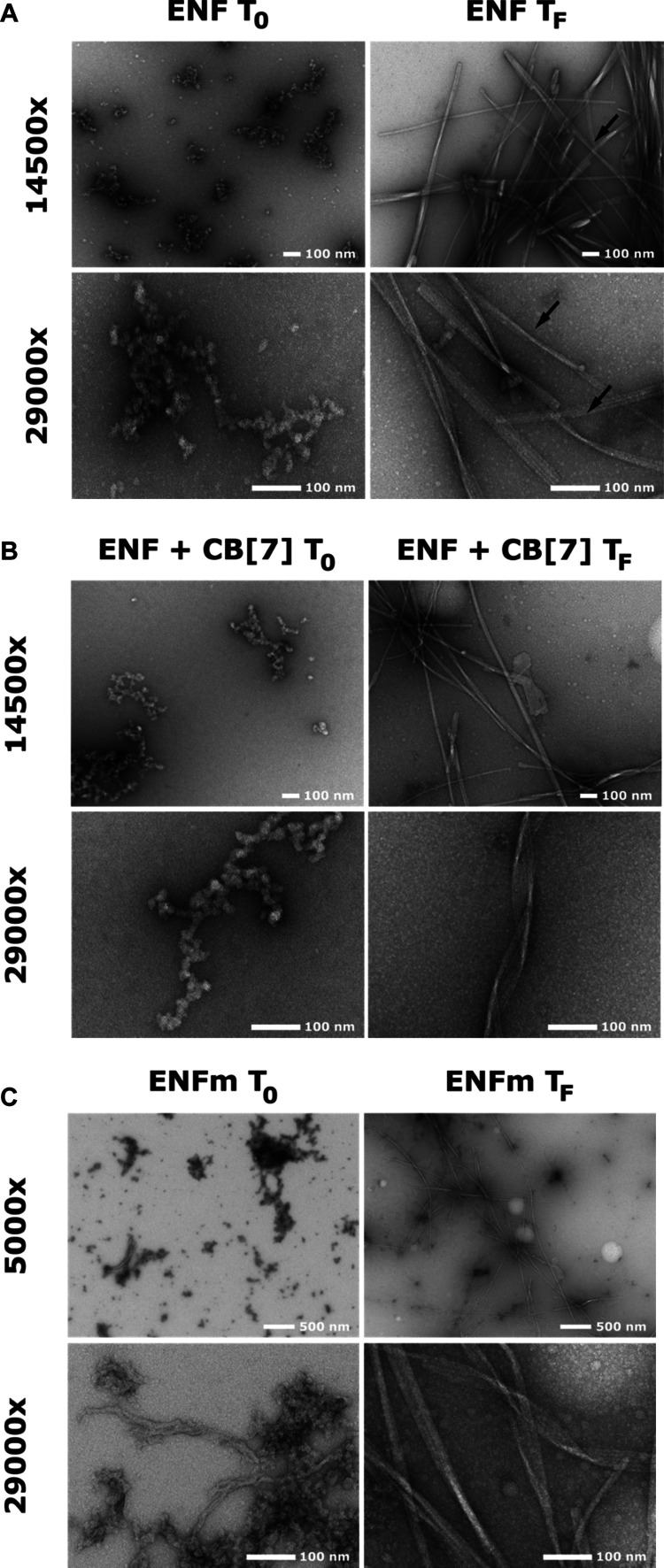
Negative-stain TEM images of ENF and ENFm peptides. (A) ENF peptide at pH 6.5 and a concentration of 80 μM before (*T*_0_) and after (*T*_F_) incubation of 5 days at 37 °C. (B) ENF peptide with an equimolar concentration of CB[7] prior (*T*_0_) and after (*T*_F_) incubation for a period of 45 h at 37 °C. Other conditions are as for (A). (C) ENFm peptide prior (*T*_0_) and after (*T*_F_) incubation for a period of 45 h at 37 °C. Other conditions are as for (A). Magnifications are indicated on the left hand sides of each panel.

Motivated by results from ThT-assays and CD spectroscopy, transmission electron microscopy (TEM) was used to image samples of ENF after finalization of ThT-assays in an attempt to confirm the existence of mature fibrils and describe morphological features. Figure 4A shows TEM images taken before (ENF *T*_0_) and after (ENF *T*_F_) a ThT-assay at pH 6.5. Oligomeric species of varying sizes between 20 to 300 nm are visible in freshly prepared samples of ENF, indicating self-association prior to ThT-assay incubation. No fibrils are apparent, but oligomers can be observed, appearing to consist of chained globular units displaying varying degrees of branching. At the end of the 120 h incubation period, TEM images of ENF show numerous, well-developed fibrillar structures. Mature fibrils shown here are ribbon-like, predominantly straight, exhibit a commonly observed characteristic twist (black arrows), have a width/height of 20–30 nm, and are up to a few μm long. These results confirm the presence of fibrils, corroborating observations made by other methods.

### Binding of CB[7] to ENF Characterized by ITC

CB[7] has previously been shown to fully inhibit fibrillation of insulin by binding predominantly to exposed N-terminal Phe residues.^21^ Aromatic amino acid residues are believed to play an important role in the fibrillation process, stabilizing fibril architecture through π-stacking interactions.^18^ ENF contains several aromatic amino acid residues in its sequence, presenting potential guest molecules which could be harbored within the hydrophobic cavity of CB[7] (N-terminal Tyr, 3 interchain Trp, C-terminal Phe residue). Reported binding affinities for these amino acids are in the micromolar (Phe) to millimolar range (Trp and Tyr).^39,40^

**Figure 5:**
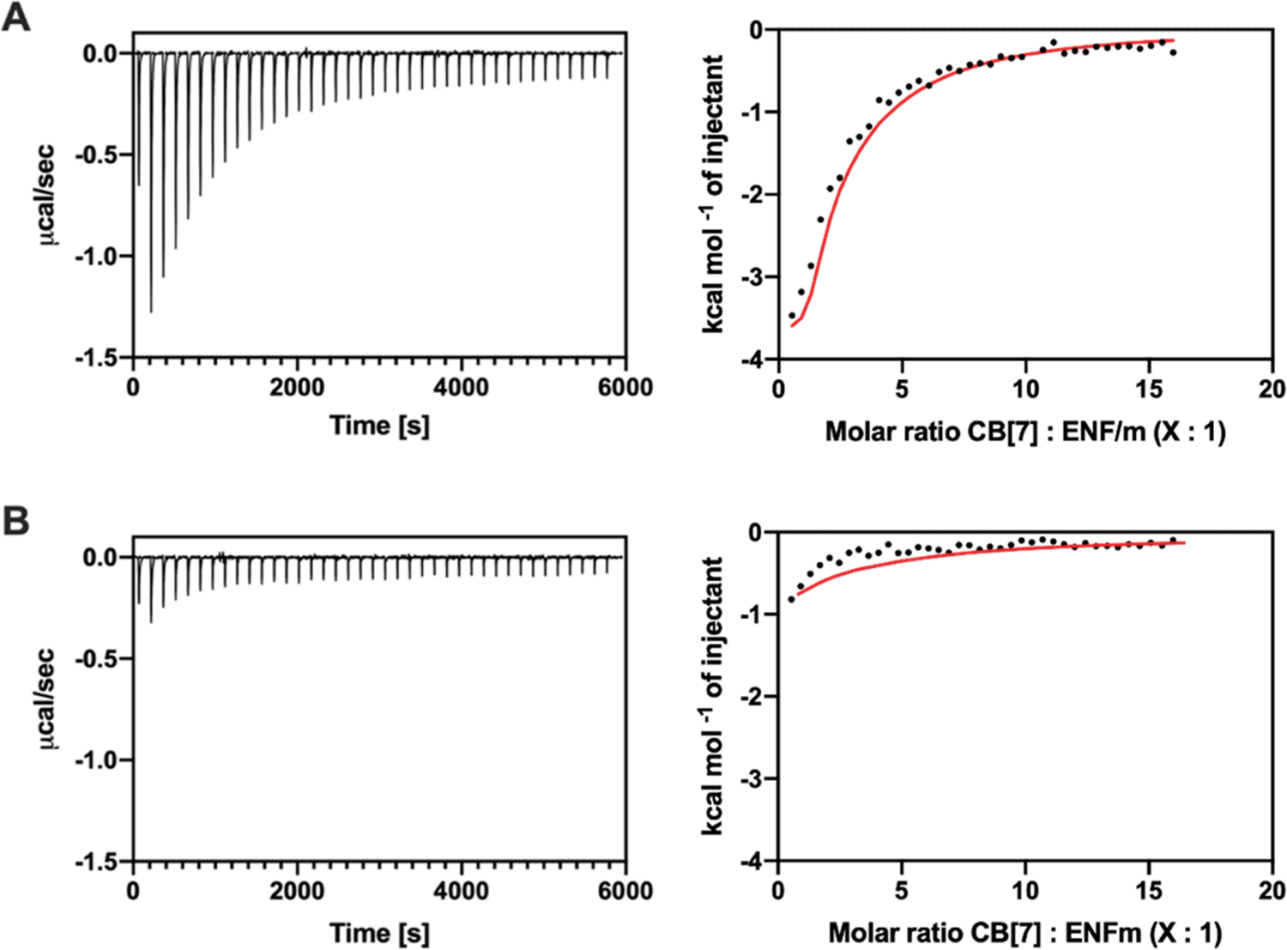
Isothermal titration calorimetry for ENF/CB[7] and ENFm/CB[7] measured at 25 °C. The first of a total of 40 injections was discarded, and changes of heat arising from dilution of the titrant were subtracted from results. (A) Thermogram (left panel) and integrated changes of heat with fitted isotherm (right panel) of a 2 mM solution of CB[7] into a 100 μM solution of ENF. Fitting was achieved via a one-set of sites model. (B) Thermogram (left panel) and integrated changes of heat with fitted isotherm (right panel) of a 2 mM solution of CB[7] into a 100 μM solution of ENFm. Fitting was achieved via a one-set of sites model.

Isothermal titration calorimetry was used to characterize a potential interaction of CB[7] with ENF and the single-point mutant, ENFm. The resulting thermograms and binding isotherms are shown in Figure 5. Thermodynamic parameters obtained, after fitting integrated and normalized changes in heat to a one-set of sites model, are listed in Table 2.

**Table 2:** Stoichiometry and Thermodynamic Parameters Obtained from Titration of ENF and ENFm with CB[7]

	*N*	*K*_a_ [M^–1^]	Δ*H* [kcal/mol]	–*T*Δ*S* [kcal/mol]
ENF	2.2 ± 0.3	2.4 ± 0.2 × 10^5^	–3.7 ± 0.3	–3.64 ± 0.13
ENFm	1.3 ± 0.1	2.8 ± 0.5 × 10^3^	–5.4 ± 0.5	0.70 ± 0.02

As CB[7] is titrated into a solution of ENF, heat is consumed resulting in endothermic peaks, which decrease in size as the titration progresses (Figure 5A). The calculated apparent binding affinity is in the micromolar range, comparable to values obtained previously for the titration of Phe by CB[7]. The thermodynamic parameters also indicate that the binding of CB[7] is entropically driven. The increase in entropy is likely attributable to displacement of high-energy water molecules from the CB[7] cavity as well as release of the hydration shell around the C-terminal Phe residue upon formation of a CB[7]-Phe inclusion complex. The stoichiometry (N) suggests the presence of at least two binding sites. In addition to the C-terminal Phe, adjacent Trp or the N-terminal Tyr could represent other potential interaction sites for CB[7].

Given the micromolar binding affinity of CB[7] to Phe, a mutant ENFm with the C-terminal Phe residue exchanged for Ala was also tested for its interaction with CB[7] by ITC in order to identify this residue as the specific binding site. The corresponding thermogram and binding isotherm are shown in Figure 5B. The changes in heat observed in the ENFm/CB[7] thermogram are significantly lower than those for ENF/CB[7]. The resulting binding isotherm yields a stoichiometry of ∼1 and an apparent millimolar binding affinity when fitted with a one-set of sites model, demonstrating the weaker interactions between ENFm and CB[7] compared to ENF and CB[7].

The reduced interaction observed for ENFm indicates that the specific binding site for CB[7] in ENF is the C-terminal Phe residue.

### Fibrillation of ENF in the Presence of CB[7]

**Figure 6:**
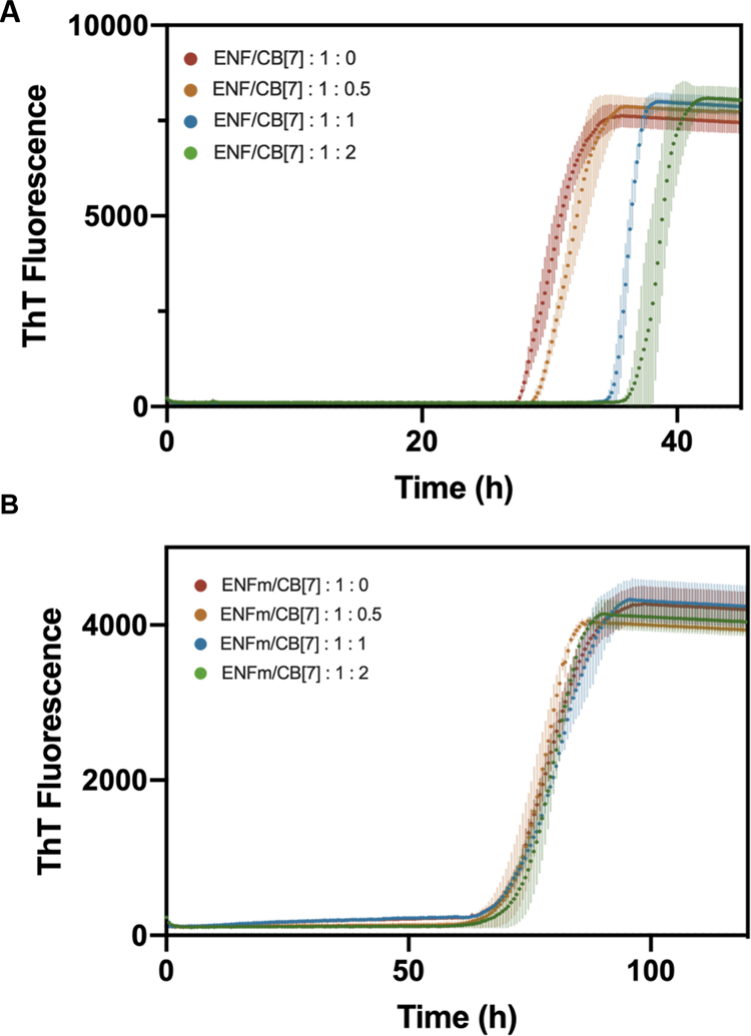
ThT fluorescence of ENF and ENFm at increasing ratios of CB[7]. (A) ENF, samples were incubated for a total of 45 h at 37 °C. ThT profiles were fitted to eq 1 with calculated kinetic parameters being listed in Table 3. (B) ENFm, samples were run for a total of 120 h at 37 °C. ThT profiles were fitted with eq 1 with calculated kinetic parameters being listed in Table 4.

ThT-assays were subsequently run for ENF in the presence of CB[7] to examine whether fibrillation could be inhibited. ThT-assays were performed at previously selected conditions (pH 6.5, 80 μM) in the absence of CB[7] and at increasing molar ratios of 1 to 0.5, 1, and 2 (ENF: CB[7]) and are shown in Figure 6A. Kinetic parameters of *t*_lag_ and *k*_app_ are listed in Table 3.

**Table 3:** Lag Time (*t*_lag_) and Apparent Growth Rate (*k*_app_) of ENF at Increasing CB[7] Concentrations

	*t*_lag_ [h]	*k*_app_ [h^–1^]
ENF	28.1 (±0.4)	0.9 (±0.02)
ENF/CB[7] (1:0.5)	29.6 (±0.3)	1.0 (±0.3)
ENF/CB[7] (1:1)	35.3 (±0.3)	2.3 (±0.2)
ENF/CB[7] (1:2)	37.4 (±1.0)	2.1 (±0.9)

At a molar ratio of 1:0.5 (ENF/CB[7]), a small increase in Fl_max_ can be seen with a slight shift to longer *t*_lag_ values, while *k*_app_ remains comparable to values recorded in the absence of CB[7]. At equimolar ratios of ENF and CB[7], *t*_lag_ increases by 26% compared to ThT profiles of ENF without CB[7] and increases further, although not as visibly at a molar ratio of 1:2 (33% more than at 1:0 ratio).

CD spectra of ENF in the presence of CB[7] were also obtained before and during ThT-assay incubation, in order to follow changes in the secondary structure. Figure S2B shows spectra recorded in the presence of equimolar concentrations of ENF and CB[7] before and after 15, 30, and 45 h of incubation at 37 °C. Spectra recorded prior to incubation in the presence of CB[7] are comparable to those recorded for ENF alone, with no clear solution structure for freshly prepared samples (Figure S2B, red bars). Visible minima emerge in the range between 215 and 220 nm as incubation progresses, characteristic of β-sheet contributions to the overall structure. Spectra recorded at time points during incubation show less changes from the spectra prior to incubation compared to those observed in the absence of CB[7], suggesting a delay in random coil to β-sheet structure transition.

Secondary structure contributions, obtained from deconvolution using the BeStSel server, are plotted in Figure 3B. Similar to ENF in the absence of CB[7], ENF in the presence of CB[7] is predominantly unstructured in solution (46.3%) and transitions toward increasing contributions from the β-sheet structure. However, compared to secondary structure contribution values obtained in the absence of CB[7], β-sheet structure contribution after incubation only reaches 27.9% (compared to 73.4% in the absence of CB[7]). Simultaneously, contributions arising from α-helical motifs remain around 20% up to 30 h before falling to 0% after incubation for 45 h.

TEM images before and after incubation were also taken for ENF in the presence of CB[7] (Figure 4B). They show the presence of oligomeric species in freshly prepared samples (*T*_0_); similar to images taken in the absence of CB[7], no fibrillar structures are visible here. In contrast to the ribbon-like fibrils observed after 45 h incubation for ENF alone, the presence of CB[7] results in tightly twisted fibrils for ENF that exhibit a visibly shorter pitch. In general, changes in ThT fluorescence and CD spectra suggest the presence of mature fibrils rich in the β-sheet secondary structure, which the corresponding TEM images show to be of long, straight, and tightly twisted morphology. These results indicate that CB[7] is unable to fully prevent fibrillation of ENF but induces a delay of the fibrillation process when added at equimolar ratios. In addition, the presence of CB[7] appears to yield a change in ENF fibril morphology, hinting at differing fibrillation pathways compared to those formed with ENF alone.

### Fibrillation of ENFm

Fibrillation profiles of ENFm (which lacks Phe36, the proposed target residue for CB[7]) were recorded to examine how the substitution of the C-terminal aromatic acid residue affects kinetic parameters and morphological features. Given that CB[7] delayed the onset of fibrillation in solutions containing ENF, CB[7] was also added to solutions of ENFm to investigate if a delay in fibrillation is also observable in the absence of the proposed primary binding site at the C-terminus.

Figure 6B shows ThT profiles of ENFm in the absence and at increasing molar ratios of CB[7]. ThT-assays were performed under identical solution conditions (pH 6.5, 80 μM ENFm concentration and 37 °C) and ran for a total period of 120 h. Fl_max_ measured for ENFm is significantly lower compared to Fl_max_ for ENF in identically measured samples. Furthermore, the *t*_lag_ values listed in Table 4 are more than twice as long as *t*_lag_ values obtained for ENF. Addition of CB[7] up to a molar ratio of 1:2 (ENFm/CB[7]) appears to have no effect on either *t*_lag_ or on measured Fl_max_. Lastly, *k*_app_ values are not impacted by the addition of CB[7] and are significantly lower than those obtained for ENF but comparable to those observed for ENF/CB[7].

**Table 4:** Lag Time (*t*_lag_) and Apparent Growth Rate (*k*_app_) of ENFm Fibrillation at pH 6.5

	*t*_lag_ [h]	*k*_app_ [h^–1^]
ENFm	71.2 (±0.6)	0.26 (±0.01)
ENFm/CB[7] (1:0.5)	71.6 (±2.8)	0.38 (±0.11)
ENFm/CB[7] (1:1)	72.5 (±1.6)	0.24 (±0.04)
ENFm/CB[7] (1:2)	73.5 (±2.8)	0.35 (±0.11)

In general, mutation of Phe at the C-terminus to Ala appears to prolong fibrillation onset markedly when compared to fibrillation of ENF in the presence of CB[7]. In alignment with experiments conducted for ENF, CD spectra were also recorded for ENFm before and during fibrillation (Figure S2C). Similar to ENF, ENFm appears to remain mainly unstructured in solution, with comparable secondary structure contributions calculated from deconvolution (Figure 3, red bars).

After a period of 40 h no significant change in spectra and secondary structure contributions can be seen, while spectra recorded after 80 h of incubation yield increased contributions from β-sheets. Spectra recorded after completion of 120 h incubation show a minimum in the range between 215 and 220 nm characteristic of the β-sheet structure, indicating the presence of fibrils. Deconvolution of spectra yields primarily unstructured contributions at 0 and 40 h of incubation, while β-sheets appear to form a significant part of structural motifs after 80 h reaching 43.9% after 120 h of incubation.

In the presence of CB[7], β-sheet contribution after 120 h of incubation amounts to 38.6%, slightly lower but comparable to the 43.9% observed in the absence of CB[7] (Figures S2D and S3). However, when comparing deconvoluted CD spectra after 80 h of incubation, the β-sheet contribution for ENFm reaches 45.4% while only reaching 28.9% in the presence of CB[7]. We also noted that ENFm CD spectra, both in the presence and absence of CB[7], show a visible increase in β-sheet contributions at 80 h (green bars) compared to 0 h (red bars) in Figures 3C and S3.

TEM images of ENFm were also taken before and after completion of 120 h incubation in the absence of CB[7] (Figure 4C). Similar to ENF, freshly prepared samples of ENFm show branched oligomeric structures composed of globular units. In contrast to ENF, additional wormlike structures, several hundred nanometers in length, are also present, entangled in close proximity to oligomeric species. TEM images taken after completion of the incubation period show long, straight, and twisted mature fibrils, comparable to those detected for ENF in the presence of CB[7] but different from the ribbon-like fibrils and with a longer pitch than for those observed for ENF only.

## Discussion

ENF is formulated in a sodium carbonate buffer at pH 9 to maximize solution stability during manufacture and reconstitution, before being lyophilized to yield the final drug product.^26,27^ The solubility of ENF in the present work was observed to be the highest at basic pH, with ENF solutions becoming increasingly opalescent as the pH transitioned from neutral toward mildly acidic conditions and before beginning to precipitate below pH 6. This could be explained by a steep reduction in the theoretically calculated net charge between pH 7 (net charge of −5) and pH 4 (zero net charge) (Figure S4). This is likely to increase favorable peptide–peptide interactions as the solution pH transitions toward acidic conditions. The results from the fibrillation studies presented here show a similarly strong pH dependency in that range. A previous study has suggested that protonation of a His side chain is responsible for this dependency of solution behavior in the pH range from 7 to 6.^28^

The maximum ThT fluorescence appears to be concentration-dependent. The downward trend observed at the highest concentration suggests that ThT cannot bind as effectively to fibrils at the later stages of the saturation phase. For example, the downward slope in the saturation phase might be associated with secondary pathways during fibril growth.^41^ The presence of a secondary pathway is further supported by a slower fibril growth rate at the highest concentration indicating that, after reaching the maximum ThT fluorescence, the secondary pathway dominates further fibril growth. A decrease in ThT fluorescence at high concentrations during the saturation phase has previously been observed for the fibrillation-prone peptide glucagon.^42^ The authors attributed this decrease to structural transitions in the late stages of fibril growth.^42^

The solution structure of ENF has been previously investigated, with one study examining an elongated ENF variant (NN-ENF-NITN) by FUV-CD and NMR spectroscopy, finding mainly unstructured regions and a 20% contribution of α-helical motifs extending from the middle of the sequence toward the C-terminus.^30,31^

TEM images taken of freshly prepared ENF samples reveal the presence of oligomeric species, suggesting self-association prior to incubation. ENF has been found to remain monomeric at concentrations below 20 μM but undergoes self-association above 20 μM at pH 7, resulting in a monomer–tetramer equilibrium.^30^ A further reduction in pH to 6.5 used in the present study can be assumed to result in greater propensity for self-association, as indicated by the solubility behavior observed in that pH range.

The presence of peptide oligomers is frequently observed in studies examining fibrillation. For example, glucagon is mainly monomeric at concentrations below 1 mg/mL, while self-associating into trimers above this concentration.^43^ Interestingly, this concentration-dependent equilibrium gives rise to fibrils of different morphologies, with monomeric species yielding twisted fibrils and trimeric species resulting in ribbon-like fibrils.^44^ Oligomeric species can also act as off-pathway reservoirs from which amyloidogenic species can originate via dissociation, as observed in the fibrillation of the milk protein κ-casein.^45^ In the case of ENF, oligomers could represent on-pathway precursors to nucleation and/or require dissociation into nucleation-competent species.

CB[7]’s ability to form host–guest complexes is mediated by the electronegative environment and accessibility of the putative guest residue.^46^ Residues at either end of the peptide sequence are more accessible compared to interchain Phe residues reflected by higher binding affinities for CB[7], but studies have shown that interchain Phe in peptides and even proteins can bind to CB[7] if surface exposed.^33,47^ N-terminal positioning potentiates CB[7] complexation due to ion–dipole interactions between the positively charged N-terminal amino group and the high electronegativity present on the carbonyl-lined corona of CB[7], yielding nanomolar binding affinities.^48^ We would not expect this effect in the peptides studied here, however, because the amino terminus in acetylated (Figure 1). In contrast, C-terminal positioning is less favored because the negatively charged carboxylic group results in repulsion of CB[7], resulting in much lower binding affinities in the millimolar range.^49^ In the present study, CB[7] should not only be able to bind a C-terminal (and sterically accessible) Phe residue in ENF but also experience no charge repulsion because amidation of the C-terminus neutralizes the negative charge. Binding affinities obtained in our study fit well in between these values of N- and C-terminal complexation of Phe residues in uncapped peptides.

As mentioned in the Results section, ENF/CB[7] interaction appears to be entropically driven. In addition to the release of high-energy water from the CB[7] cavity, potential dissociation of oligomeric ENF species could take place in the presence of CB[7].^50^ This form of dissociation has not been further examined or verified in the present study, but similar dissociation reactions upon CB[7] binding to a Phe and Trp containing APR within an mAb have recently been observed via fluorescence quenching experiments.^33^

A mutant lacking putative guest residues has previously been used to identify and verify binding sites for CB[7].^21,33^ ENFm, lacking the C-terminal Phe residue present in ENF, showed weaker binding affinity for CB[7] than ENF. ITC is a powerful method to determine thermodynamic parameters for well-defined binding reactions with affinities in the nM to μM range.^51^ Weak interactions, such as those observed for ENFm/CB[7], and the resulting thermodynamic parameters, have to be interpreted with caution. However, the difference in binding affinity is readily apparent when visually comparing the ENF/CB[7] and ENFm/CB[7] thermograms. The presence of two apparent binding sites in ENF is interpreted as originating from the specific binding site (i.e., Phe36) and multiple nonspecific interactions which are too weak to be resolved with ITC. A similar observation was made for entropically driven binding of several *p*-sulfonatocalixarene derivatives to Aβ42 through nonspecific and multiple hydrophobic interactions.^52^ CB[7] has recently also been proposed to bind Lys, from computational modeling studies.^22,53,54^

Macrocycles have previously been examined for their impact on peptide fibrillation.^22^ β-Cyclodextrins have been shown to bind Phe residues in Aβ, as well as binding to hydrophobic residues within the sequence of synthetic insulin derivatives, and inhibit fibrillation of these peptides.^55,56^ Similarly, in the case of insulin, *p*-sulfonatocalixarenes showed a reduction in fibrillation by binding Lys and Arg in addition to Phe residues.^22^ Moreover, *p*-sulfonatocalixarenes were not only able to inhibit fibrillation but also disintegrate amyloid fibrils.^22^

CB[7] has previously been shown to completely suppress insulin fibrillation by binding to N-terminal Phe residues.^21^ Similarly, CB[7] complexation of interchain Phe and Tyr residues in fibrillation-prone human calcitonin was found to fully inhibit the formation of mature fibrils.^34^ In general, results presented here support the observation that CB[7] prolongs the lag phase during ENF fibrillation, although not fully inhibiting it. Substitution of the C-terminal Phe residue for Ala gave rise to a significant delay in fibrillation onset but was insufficient on its own to fully inhibit the formation of mature fibrils when incubated long enough.

ENF oligomeric species remain present in solutions containing CB[7] and are comparable in size and morphology to those observed in the absence of CB[7], suggesting no apparent oligomer dissociation. Interestingly, the presence of CB[7] results in a significant change in fibril morphology, from ribbon-like (without CB[7]) to tightly twisted (with CB[7]) fibrils, hinting at the presence of an alternative fibrillation pathway mediated by interaction between the macrocycle and ENF. As discussed above, this differentiation between two alternative pathways and distinct resulting morphologies has been observed for other peptides.^42−44^ Based on the TEM images, the estimated pitch length for tightly twisted ENF fibrils is around 140 nm, corresponding to 4–5 aligned protofilaments.^57^ This distinction between ribbon-like and twisted morphologies has also been observed for α-synuclein in the presence of ThT, which was able to modulate the fibrillation pathway.^58^ In that study, the absence of ThT led to fibril heterogeneity yielding primarily tightly twisted fibrils in addition to ribbon-like fibrils, while exclusively forming ribbon-like fibrils in the presence of ThT. The authors attributed this effect to binding of ThT to species formed early on during α-synuclein fibrillation, enabling diversion to a specific fibrillation pathway and reducing fibril polymorphism observed in the absence of ThT.^58^

The presence of wormlike fibrils for ENFm prior to incubation, together with the prolongation of *t*_lag_, could indicate the existence of off-pathway fibrillation processes. In some studies wormlike morphologies have been linked to off-pathway oligomers forming on a competitive route to on-pathway oligomers formed during nucleation–polymerization.^59,60^ Off-pathway processes usually show a concentration dependency where fibrillation onset is delayed as peptide concentration is increased, as opposed to classical nucleation–polymerization processes (or on-pathway processes) where higher peptide concentrations result in shorter lag times as previously shown for glucagon-like peptide-1.^10^ This aspect of ENFm fibrillation has not been examined in the present study but could aid to verify the potential off-pathway formation of oligomers.

Upon ENFm incubation, ThT fluorescence increases but remains much lower compared to ENF and ENF/CB[7]. The observed reduction in maximum ThT fluorescence could be associated with a change in fibril architecture. However, the lower fluorescence observed for ENFm produces tightly twisted fibrils of comparable size and pitch to ENF/CB[7], where ThT fluorescence is significantly higher. The reduction in fluorescence could also stem from a reduction in hydrophobic surface patches to which ThT is able to bind. ThT is believed to bind cavities along the peptide fibrils composed of hydrophobic and aromatic amino acid side chains.^61,62^ The higher observed fluorescence in ENF/CB[7] could therefore be explained as originating from the presence of Phe36 conferring a more hydrophobic environment to ThT. Under this assumption, CB[7] could divert the ENF fibrillation pathway toward the twisted fibril morphology by binding to Phe36 but ultimately not be incorporated into ENF fibrils, explaining the higher observed ThT fluorescence.

Single-point mutations have been shown to significantly impact fibrillation kinetics and morphologies of sequences involved in pathologies such as Alzheimer’s disease and corneal stromal dystrophies.^14,63,64^ Likewise, insulin was observed to yield distinct fibrillation kinetics and morphologies as a consequence of a single amino acid substitution.^15^ At the same time, a number of different small-molecule inhibitors of peptide fibrillation have been proposed in the past.

In summary, in this study, we highlight the ability of CB[7] to modify the fibrillation kinetics of the HIV fusion inhibitor ENF. In particular, we observe fibril formation to a distinct tightly twisted morphology, which we interpret as forming from an alternative fibrillation pathway. The results show that sequestering Phe36 in ENF via CB[7] does not have the same fibrillation-delaying effect as substitution. However, we show that CB[7] is capable of reproducing comparable fibrillation morphologies, as observed for the single-point mutant ENFm. On the basis of these observations, we propose to further characterize different fibril morphologies obtained via the addition of CB[7] for ENF and, potentially, other peptides. This would be of particular interest since different fibril morphologies have been reported to exhibit distinct physicochemical properties and stabilities that have been exploited for drug delivery and biotechnological applications.^65,66^ For therapeutic applications, we are not aware of detailed toxicological studies on CB[7], although we note that it has been used as an excipient for anticancer agents in animal models, without evidence of direct toxic effects.^67^ Further, its use in reducing the toxicity of paraquat^68^ and neurotoxic drugs^69^ has been reported, suggesting potential for *in vivo* compatibility.

## Supporting Information Available

Supplementary Methods: theoretical calculation of net charge as a function of pH and pI determination; Figure S1: ThT-assay of ENF at physiological pH (7.4) at concentrations of 50, 75, and 100 μM; Figure S2: CD spectra of ENF and ENTm peptides; Figure S3: secondary structure contributions in ENFm in the presence of CB[7] obtained by deconvolution using the BeStSel webtool; and Figure S4: titration curve of theoretical ENF net charge from pH 1 to 10 (PDF)

## Notes

For the purpose of open access, the author has applied a Creative Commons Attribution (CC BY) licence (where permitted by UKRI, “Open Government Licence” or “Creative Commons Attribution No-Derivatives (CC BY-ND) Licence” may be stated instead) to any Author Accepted Manuscript version arising.
