# Supplementary material for: Modulation of the Fibrillation Kinetics and Morphology of a Therapeutic Peptide by Cucurbit[7]uril
Source: Mol Pharm. 2023 Jun 16;20(7):3559–69. doi: 10.1021/acs.molpharmaceut.3c00185 (PMC10324397; doi:10.1021/acs.molpharmaceut.3c00185)
Supplement: Supplementary file 1 — mp3c00185_si_001.pdf [file mp3c00185_si_001.pdf]

Supplementary Materials

Supplementary Methods

Theoretical calculation of net charge as a function of pH and pI determination

The isoelectric point at which the net charge of a protein or peptide is zero was determined by calculating the net charge of ENF at different pH values using the Henderson – Hasselbalch relationship. Contributions of ionizable residues was calculated as follows:

For negatively charged residues:

$$\sum_{i=1}^{n}\frac{-1}{1+\ 10^{pkn-pH}}\#(S4.1)$$

with pkn representing the acid dissociation constant of the negatively charged side chain and for positively charged residues:

$$\sum_{i=1}^{n}\frac{1}{1+\ 10^{pH-pkp}}\#(S4.2)$$

with pkp representing the acid dissociation constant of the positively charged side chain. The net charge is then obtained by forming the sum of contributions. Pka values of amino acid side chains used for the calculation of net charge were 10.517 (Lys), 12.503 (Arg), 6.018 (His), 4.317 (Glu) and 3.887 (Asp)[1].

Supplementary Figures

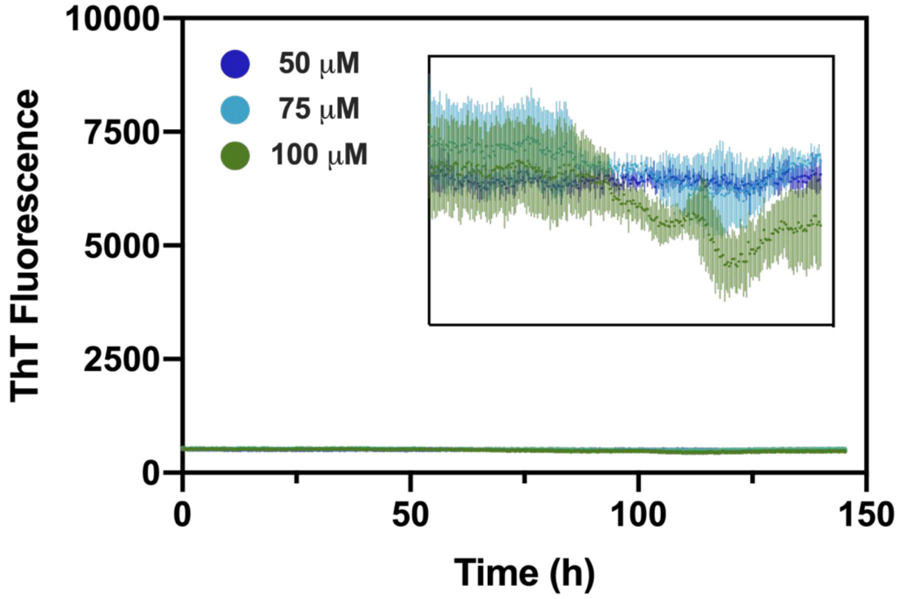

Figure S1. ThT – assay of ENF at physiological pH (7.4) at concentrations of 50, 75 and 100 μM. Magnification of plot in inset.

A

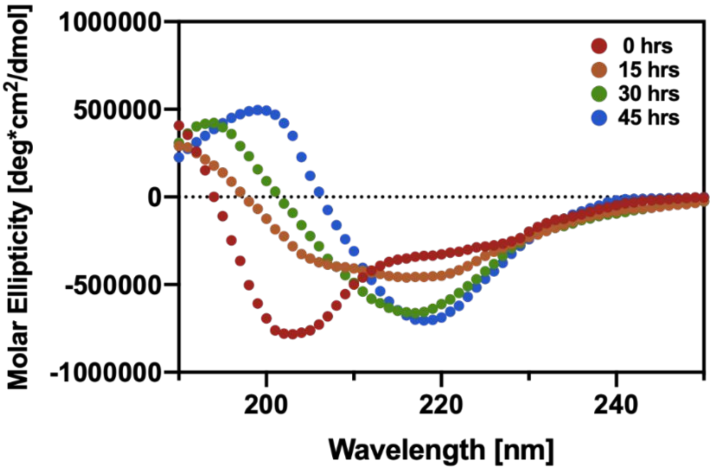

B

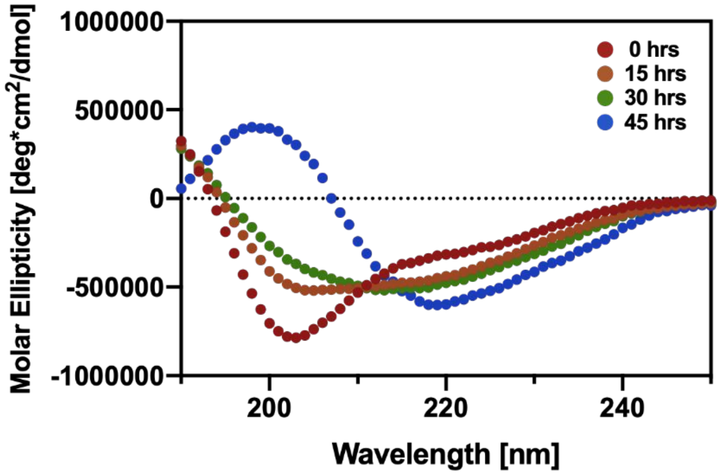

C

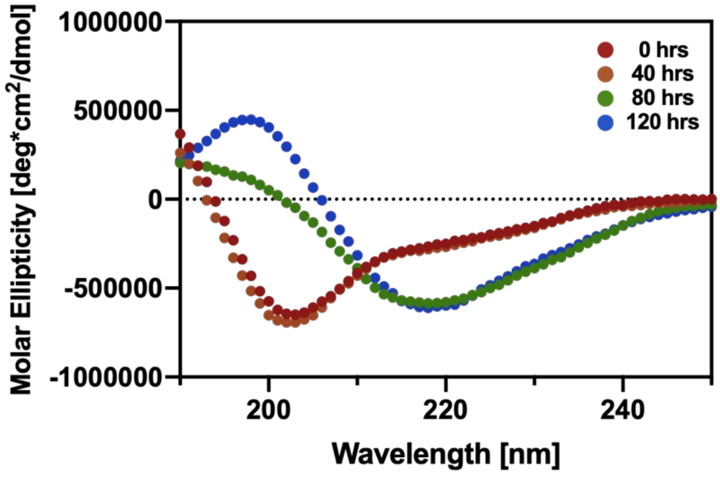

D

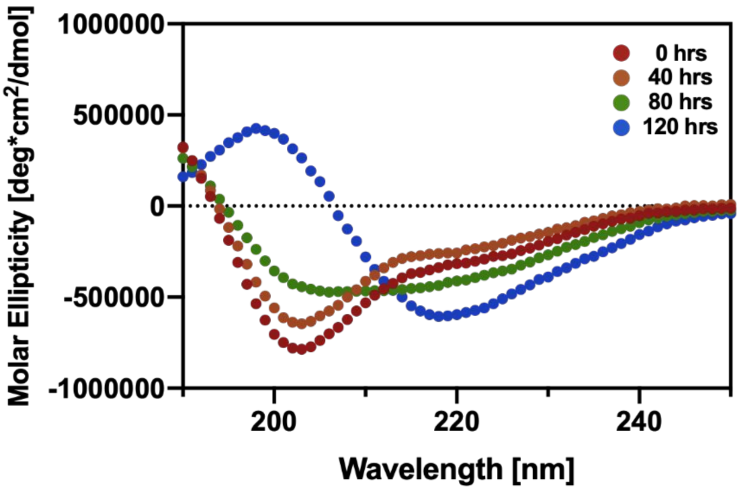

Figure S2. CD spectra of ENF and ENTm peptides. A blank spectrum containing buffer was subtracted from each recorded ENF spectra and curves were smoothed. A) CD spectra of ENF before and after 15, 30 and 45 hrs of incubation at 37° C. B) CD spectra of ENF in presence of CB[7] recorded before and during a 45 hrs incubation at 37° C. C) CD spectra recorded for ENFm at different timepoints of a 120 hr incubation. ENFm was incubated at a concentration of 80 μM at 37° C with CD spectra recorded at 25 °C. D) CD – spectra of ENFm in presence of CB[7] measured at 25 °C. Spectra were recorded of samples prior to a ThT – assay as well as after 40, 80 and 120 hrs of incubation at 37°C.

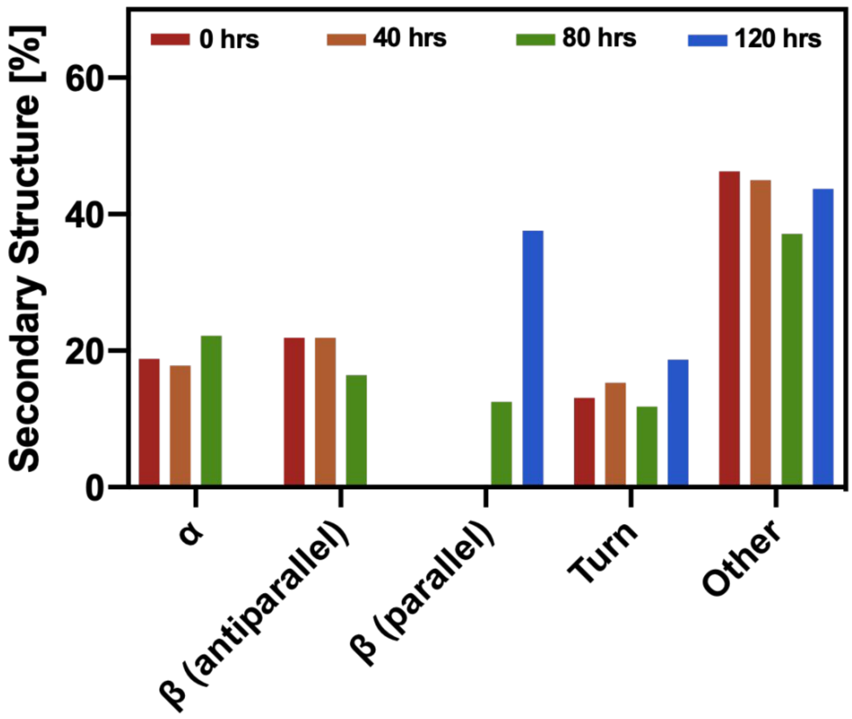

Figure S3. Secondary structure contributions in ENFm in presence of CB[7] obtained by deconvolution using the BeStSel webtool.

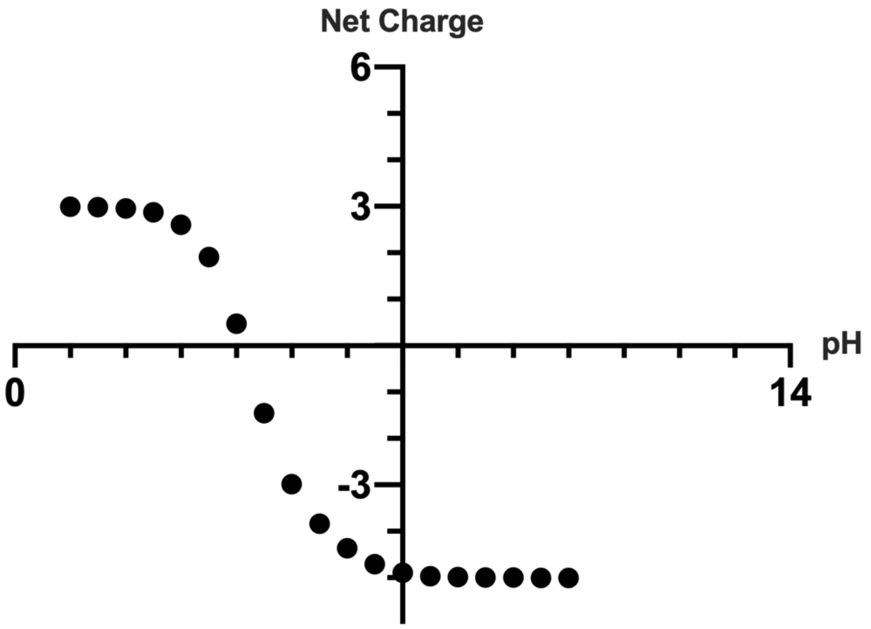

Figure S4. Titration curve of theoretical ENF net charge from pH 1 to 10. The pI of ENF where the net charge is zero is approximately at pH 4. Calculations were made via the Henderson – Hasselbalch equations (see supplementary methods)[1].

References

1. Kozlowski, L. P. IPC – Isoelectric Point Calculator. *Biol. Direct* **11**, 55 (2016).
